# Challenges and Opportunities of γδ T Cell-Based Immunotherapy for Glioblastoma

**DOI:** 10.3390/biomedicines14081770

**Published:** 2026-08-06

**Authors:** Chun-Chieh Chao, Hsieh-Tsung Ethan Shen, Bo-Xiang Benjamin Zhang, Ting-Hsuan Collette Chao, Ching-Dong William Wang, Chung-Che Wu

**Affiliations:** 1 Graduate Institute of Injury Prevention and Control, College of Public Health, Taipei Medical University, Taipei City 235057, Taiwan; 2 Department of Emergency Medicine, Taipei Medical University Hospital, Taipei City 11031, Taiwan; 3 Department of Emergency Medicine, School of Medicine, College of Medicine, Taipei Medical University, Taipei City 235057, Taiwan; 4 Ji Yan Biomedical (JY BioMed) Co., Ltd., Taipei City 11561, Taiwan; 5 Division of Clinical Cariology and Endodontology, Department of Oral Rehabilitation, School of Dentistry, Health Sciences University of Hokkaido, Tobetsu 061-0293, Japan; 6 SL Science Holding Ltd., Taipei City 11560, Taiwan; 7 Ph.D. Program in Medical Neuroscience, College of Medical Science and Technology, Taipei Medical University and National Health Research Institutes, Taipei City 11031, Taiwan; 8 Department of Molecular and Cell Biology, College of Letter and Science, University of California, Berkeley, CA 94720, USA; 9 Department of Neurosurgery, Taipei Medical University Hospital, Taipei City 11031, Taiwan; 10 Department of Surgery, School of Medicine, College of Medicine, Taipei Medical University, Taipei City 235057, Taiwan

**Keywords:** glioblastoma, γδ T cells, Vγ9Vδ2, Vδ1, NKG2D, off-the-shelf cellular immunotherapy, tumour microenvironment, adoptive cell therapy, tumour hypoxia, interleukin-17

## Abstract

Glioblastoma remains the most lethal primary malignancy of the central nervous system, and the modest gains achieved with maximal surgery, radiotherapy and temozolomide have not been matched by the immune checkpoint inhibitors and antigen-specific vaccines that reshaped the treatment of many extracranial cancers. The recurrent disappointment of these approaches has been attributed less to a single molecular lesion than to a confluence of obstacles: profound intratumoural heterogeneity, a densely immunosuppressive and myeloid-rich microenvironment, sequestration and exhaustion of conventional T cells, and the practical difficulty of delivering effectors across the blood–brain barrier. Against this background, γδ T cells have attracted interest as an unconventional effector population that recognises transformed cells through stress-associated and metabolic cues rather than peptide–major histocompatibility complex (MHC) complexes, that kills in an MHC-unrestricted manner, and that can be expanded from healthy donors for allogeneic, off-the-shelf use with little expectation of graft-versus-host disease. This narrative review examines, with a deliberately critical lens, the biological rationale and the experimental evidence for γδ T cell-based immunotherapy of glioblastoma. We summarise the developmental biology and functional subsets of human γδ T cells, the natural killer group 2 member D (NKG2D)-, DNAX accessory molecule 1 (DNAM-1)- and T-cell-receptor-dependent mechanisms through which they engage glioblastoma cells and glioma stem-like cells, and the in vitro and animal-model studies that underpin the field, taking care not to overstate efficacy that has so far been demonstrated only in preclinical or early-phase settings. We then weigh the principal opportunities—locoregional and repeated dosing, combination with chemoradiotherapy, checkpoint blockade and antibody-based redirection—against barriers that include limited persistence, uncertain intratumoural trafficking, donor and manufacturing variability, and the unsettled requirements of potency testing and trial design. We give particular weight to what becomes of γδ T cells inside a hostile tumour—the exhaustion-like dysfunction that follows chronic stimulation, the oxygen and glucose dependence of their effector programme, the interleukin-17-polarising signals generated by activated microglia and by genotoxic therapy, and the confounding effect of corticosteroids—together with the engineering and pharmacological strategies proposed to counter them. The first peer-reviewed phase 1 report of intracranially delivered, drug-resistant γδ T cells has now appeared and documents tolerability in a small, single-arm cohort without establishing survival benefit. Throughout, γδ T cells are presented as a biologically plausible but still investigational strategy whose clinical value will be determined by adequately powered trials rather than by mechanistic appeal alone.

## 1. Introduction

Glioblastoma, designated a grade 4 astrocytic tumour in the 2021 World Health Organization classification of central nervous system neoplasms [1], is at once uncommon in incidence yet profound in clinical impact. As the most frequent malignant primary brain tumour in adults, with an incidence of roughly three cases per 100,000 person-years, it presents a burden driven by near-universal recurrence and limited survival [2]. The therapeutic standard established two decades ago—maximal safe resection followed by radiotherapy with concurrent and adjuvant temozolomide—represented a landmark advance and continues to provide the foundation of first-line care; at the same time, the proportion of patients alive at five years shows limited modest improvement in the defining randomised trial and its long-term analysis [3,4]. The subsequent addition of alternating electric tumour-treating fields produced a statistically significant and yet clinically incremental survival gain [5]. The recent research achievements have shaped modern glioblastoma management and should be understood not as evidences of failure, but as an intensified call for research with combined efforts across disciplines.

Several features of the disease help explain the need of further innovation and define what an effective intervention must accomplish. Recurrence is almost universal and typically occurs within two centimetres of the original resection margin, often within infiltrated tissue that may not be frankly enhancing, so that local control of the visible tumour does not equate to disease control [4]. Molecular factors such as methylation of the O6-methylguanine-DNA methyltransferase promoter modulate temozolomide benefit yet they do not fully resolve the biological complexity underlying treatment resistance. In addition, no predictive biomarker reliably identifies the minority of long-term survivors. The cumulative lesson of three decades of incremental progress is that single-target, single-mechanism strategies may be less likely to remedy a tumour defined by plasticity and redundancy. This has shifted conceptual interest toward effectors capable of recognising malignancy through several independent, antigen-agnostic routes—properties that are characteristic of innate-like lymphocytes such as γδ T cells [6] and that frame the rationale examined in the remainder of this review.

Immunotherapy was a natural candidate because of its transformative effect in melanoma, lung cancer and several haematological malignancies. In glioblastoma, the translation has been challenging and highly informative. The phase 3 CheckMate 143 trial found no overall-survival advantage for the programmed cell death protein 1 (PD-1) inhibitor nivolumab over bevacizumab in recurrent disease [7]; the EGFRvIII peptide vaccine rindopepimut, despite a compelling antigen-specific rationale, did not improve survival in the phase 3 ACT IV study, with antigen loss emerging as an important biological lesson [8]; and a single infusion of EGFRvIII-directed chimeric antigen receptor (CAR) T cells, while reaching the tumour and engaging antigen, provoked adaptive mechanisms such as antigen modulation and adaptive resistance [9]. These outcomes are not random misfortune. Rather, they reflect a set of structural impediments—antigenic heterogeneity, an actively immunosuppressive microenvironment, anatomical sequestration of effector cells and a partially intact blood–brain barrier [10,11]. These efforts have refined the roadmap for further therapies and strengthened therapies for approaches that are less dependent on a single antigen or conventional peptide–MHC recognition.

If the limiting factor is frequently the quality and accessibility of the effector compartment rather than the absence of a target, then effector populations that operate outside the conventional peptide–MHC paradigm merit attention. γδ T cells occupy precisely this niche. They constitute a minor fraction of circulating lymphocytes but bridge innate and adaptive immunity, sensing markers of cellular stress and metabolic dysregulation that are broadly shared across transformed cells [12,13]. Their recognition of tumour cells does not depend on a defined surface antigen or on a particular human leukocyte antigen (HLA) type, and healthy-donor γδ cells can be expanded to clinical scale for allogeneic use without the alloreactivity that complicates conventional T-cell transfer [14,15]. These properties are individually attractive for a tumour as heterogeneous and immunoprivileged as glioblastoma, and collectively they have generated a research programme spanning early in vitro observations, animal models and first-in-human trials. This review evaluates that programme. We set out the relevant biology of glioblastoma and of γδ T cells, assess the preclinical and emerging clinical evidence, and identify where the genuine opportunities and the practical barriers where additional academic efforts may have meaningful impact. A note on terminology is warranted before proceeding. We use glioblastoma for the isocitrate dehydrogenase-wild-type grade 4 astrocytoma defined in the 2021 World Health Organization classification [1], and reserve glioma for statements whose supporting evidence extends beyond glioblastoma—glioma cell lines of mixed provenance, high-grade glioma cohorts that include grade 3 tumours, and murine glioma models. The compound terms glioma stem-like cell and high-grade glioma are retained throughout because they are the established usage in the cited work.

## 2. Biological Characteristics of Glioblastoma Relevant to Immunotherapy

An informed appraisal of any immunotherapy for glioblastoma must begin with the tumour features that have repeatedly blunted immune attack. Four interlocking characteristics are most consequential: heterogeneity, microenvironmental immunosuppression, the barriers imposed by the central nervous system, and the dysfunction of the host T-cell compartment.

### 2.1. Tumour Heterogeneity and Antigen Escape

Glioblastoma is heterogeneous at every scale. Bulk transcriptomic profiling resolved the disease into molecular subtypes with distinct genetic drivers [16], but single-cell analyses subsequently showed that these subtypes coexist within individual tumours, with malignant cells distributed across interconvertible neural-progenitor-like, oligodendrocyte-progenitor-like, astrocyte-like and mesenchymal-like states whose proportions are shaped by genetics and microenvironmental cues [17,18]. Superimposed on this plasticity is a hierarchy of glioma stem-like cells that sustain tumour growth, resist radiation through preferential activation of the DNA-damage response, and repopulate the tumour after cytotoxic therapy [19,20,21]. For immunotherapy, the implication is direct and unforgiving: any strategy keyed to a single antigen invites the outgrowth of antigen-negative variants, a failure mode demonstrated clinically when EGFRvIII was lost after vaccination and after CAR-T infusion [8,9]. Approaches that recognise multiple or antigen-independent cues are therefore conceptually better matched to the disease, and this argument recurs throughout the rationale for γδ T-cell therapy.

### 2.2. The Immunosuppressive Microenvironment

The glioblastoma microenvironment is not merely indifferent to immune effectors but actively hostile to them. Tumour-associated macrophages and microglia can constitute up to half of the tumour mass and are polarised toward phenotypes that support invasion and suppress cytotoxic responses [22,23]. Regulatory T cells accumulate within tumours and carry prognostic weight [24], while metabolic competition, hypoxia and inhibitory ligands further constrain effector function [22]. This milieu does more than dampen incoming effectors; it shapes which therapeutic strategies are plausible, favouring cells that retain function under suppressive conditions or that are delivered in numbers and at sites that partly bypass systemic regulation. The same suppressive circuitry is one reason that checkpoint blockade alone, which depends on reinvigorating a pre-existing T-cell response, has underperformed in this disease [7,10].

The composition of this immunosuppressive compartment is itself instructive. The myeloid cells that dominate the glioblastoma microenvironment derive from both blood-borne monocytes and resident microglia, and their relative contributions and phenotypes shift with tumour location, genetic subtype and treatment [23]. These cells do more than occupy space: they remodel the metabolic environment, depleting nutrients and generating immunosuppressive metabolites, and they reinforce regulatory T-cell function [22,24]. For an adoptively transferred effector, this has a concrete consequence—activity demonstrated in nutrient-replete culture may not survive transfer into a hypoxic, metabolically hostile tumour. Whether γδ cells, with their innate-like metabolic flexibility, are more resistant to these constraints than conventional T cells is an open and testable question rather than an established advantage, and it is one that in vitro cytotoxicity assays are poorly designed to answer; the evidence available so far, discussed in Section 7.2, gives more grounds for caution than for optimism [25].

Microglia are not merely suppressive bystanders in this compartment but active instructors of γδ phenotype, a point of direct relevance to any γδ-based strategy in the brain. In co-culture systems, supernatants from microglia stimulated through Toll-like receptors 2, 4, 7 or 9 activated γδ T cells by way of interleukin-1β and interleukin-23 and polarised them towards an interleukin-17-producing, neurotoxic phenotype; the effect required myeloid differentiation primary response 88 signalling and, for neuronal injury, direct contact between γδ cells and neurons [26]. That work was performed in a neuroinflammatory rather than a tumour context, and its extrapolation to glioblastoma is an inference rather than a demonstration. The ingredients are nonetheless all present in the treated glioblastoma bed, where surgery and irradiation liberate damage-associated signals into a compartment dominated by microglia and tumour-associated macrophages [22,23]. Two implications follow for therapy: the local myeloid milieu may actively steer both endogenous and infused γδ cells away from the cytotoxic type 1 programme on which the therapeutic rationale depends, and interleukin-17 polarisation is not a neutral outcome in the central nervous system. We return to this axis in Section 7.3 and Section 9.2.

### 2.3. The Blood–Brain Barrier and CNS Immune Specialisation

The central nervous system was long regarded as immunologically privileged, a view refined rather than overturned by the rediscovery of meningeal lymphatic vessels and a more nuanced understanding of CNS–immune communication [27]. For therapeutic purposes, the practical issue is access. The blood–brain barrier is heterogeneously, and often incompletely, disrupted in glioblastoma; contrast enhancement marks regions of breakdown, but infiltrative tumour beyond the enhancing margin frequently lies behind a relatively intact barrier [28]. Systemically administered cells and antibodies may therefore reach the bulk tumour while sparing the invasive front from which recurrence arises. This consideration has driven interest in locoregional delivery and has direct bearing on how γδ T cells, like other effectors, should be administered.

### 2.4. T-Cell Dysfunction and Sequestration

Even when conventional T cells are mobilised, glioblastoma compromises them in characteristic ways. Tumour-infiltrating CD8 T cells in glioblastoma display an exhaustion signature that is unusually severe relative to other tumour types, with broad co-expression of inhibitory receptors [29]. Beyond intratumoural exhaustion, naïve T cells are sequestered in the bone marrow in the setting of intracranial tumours through loss of surface sphingosine-1-phosphate receptor 1, producing a systemic T-cell lymphopenia that limits the pool available for any T-cell-dependent therapy [30]. These host-side deficits help explain why αβ-T-cell-centred strategies have struggled and provide part of the rationale for supplying fresh, externally manufactured effectors—an argument that applies to allogeneic γδ cells as much as to engineered αβ products [10,11].

## 3. Overview of γδ T Cells

γδ T cells are defined by a T-cell receptor (TCR) composed of γ and δ chains and represent roughly one to ten per cent of circulating T cells, with substantial enrichment in epithelial and mucosal tissues [12,31]. Functionally they straddle the innate-adaptive divide: they respond rapidly and without the clonal expansion kinetics of conventional T cells, yet they rearrange a somatically diversified receptor. Their physiological role has been framed as lymphoid stress surveillance—the detection of dysregulated, infected or transformed cells through conserved stress cues rather than through foreign peptides [13]. The principal human subsets are distinguished by their δ-chain usage and by their anatomic distribution and ligands (Table 1).

### 3.1. Vγ9Vδ2 Cells

The dominant subset in adult human blood pairs a Vγ9 chain with Vδ2 and is activated by small non-peptidic phosphoantigens, most importantly the isoprenoid intermediate (E)-4-hydroxy-3-methyl-but-2-enyl pyrophosphate of microbial origin and the endogenous isopentenyl pyrophosphate that accumulates when the mevalonate pathway is dysregulated, as it commonly is in transformed cells [32]. Phosphoantigen sensing is not direct TCR-ligand binding in the classical sense but an inside-out mechanism mediated by butyrophilin 3A1, whose intracellular B30.2 domain binds phosphoantigen [33,34], in obligate partnership with butyrophilin 2A1, which engages germline-encoded regions of the Vγ9 chain [35,36]. Because aminobisphosphonates such as zoledronate raise intracellular phosphoantigen levels, they both sensitise target cells and drive selective ex vivo expansion of Vγ9Vδ2 cells, the basis for most clinical manufacturing protocols [37,38,39]. This metabolic mode of recognition is attractive for glioblastoma precisely because it is antigen-agnostic and keyed to a hallmark of malignancy rather than to a lineage marker.

### 3.2. Vδ1 and Other Subsets

Vδ1 cells predominate in epithelia, gut and, after differentiation, in peripheral blood, and they are not restricted to phosphoantigen recognition; they respond to stress-induced self-ligands and to lipid antigens presented by CD1 molecules, and they show adaptive-like clonal expansion in response to persistent challenge [15,31]. Their tissue tropism and relative resistance to activation-induced death have made them an attractive substrate for engineered, off-the-shelf products, including CAR-modified Vδ1 cells [40]. Less abundant populations, including Vδ3 cells, contribute to the overall γδ repertoire but are less well characterised in the context of solid tumours. For glioblastoma the two subsets offer complementary attributes: the metabolic sensing and ready expandability of Vγ9Vδ2 cells, and the tissue residence and engineering tractability of Vδ1 cells.

### 3.3. Recognition and Cytotoxic Mechanisms

A property central to the therapeutic case is that γδ T cells assemble the same CD3 signalling module as αβ T cells, as recent structures of the human γδ TCR-CD3 complex confirm [41], yet they survey their environment without MHC restriction [12,42]. Their cytotoxic repertoire is correspondingly broad. Engagement of the activating receptor natural killer group 2 member D (NKG2D) by stress-inducible MHC class I-related ligands triggers TCR-independent lysis [43,44]; the DNAX accessory molecule 1 (DNAM-1) recognises the nectin-family ligands CD155 and CD112 that are frequently overexpressed on tumours [45,46]; and killing is executed through the perforin–granzyme pathway and death-receptor ligands, as shown when γδ cells eliminated colon cancer stem cells [47,48]. Beyond direct cytotoxicity, γδ cells secrete interferon-γ and tumour necrosis factor, can present antigen and licence dendritic cells, and thereby influence downstream adaptive responses [13,14]. This functional plasticity is double-edged, however: interleukin-17-producing γδ subsets can be pro-tumourigenic [49], and single-cell analyses reveal opposing cytotoxic and wound-healing programmes within tumour-infiltrating γδ populations whose balance is set partly by the expansion method [50]. Selecting and maintaining the cytotoxic phenotype is therefore an explicit manufacturing objective rather than a given.

## 4. Mechanisms Supporting the Use of γδ T Cells in Glioblastoma

**Figure 1:**
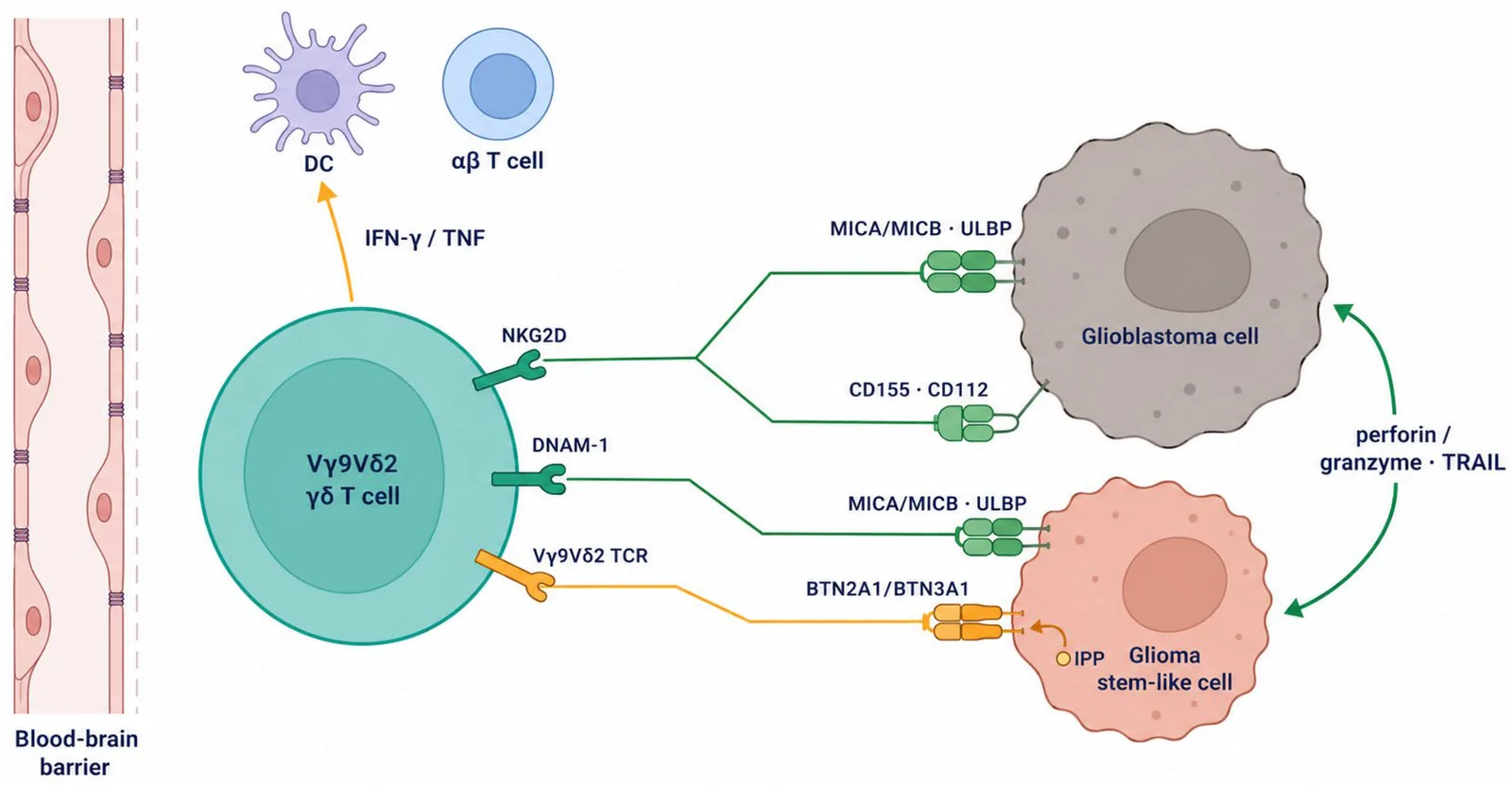
Mechanisms of γδ T-cell recognition of glioblastoma. Conceptual schematic, not to scale. A γδ T cell (teal) simultaneously surveys a glioblastoma cell (grey) and an adjacent glioma stem-like cell (coral) through three mechanistically independent, MHC-independent routes. (i) The activating receptor NKG2D engages stress-induced ligands MICA, MICB and the UL16-binding proteins (ULBPs), which are up-regulated by the DNA-damage response and are expressed on both bulk tumour and stem-like cells. (ii) DNAM-1 (CD226) engages the nectin-family ligands CD155 (PVR) and CD112 (Nectin-2). (iii) The Vγ9Vδ2 TCR senses intracellular phosphoantigen (isopentenyl pyrophosphate, IPP) accumulation—reflecting a dysregulated mevalonate pathway—via the butyrophilin BTN2A1/BTN3A1 complex (amber). Target engagement drives polarised release of perforin and granzymes and death-receptor (TRAIL-mediated) killing, while secreted interferon-γ (IFN-γ) and tumour necrosis factor (TNF) act on neighbouring dendritic cells (DC) and αβ T cells to link innate killing with adaptive immunity. The blood–brain barrier (left) is depicted as a separate delivery obstacle. Green denotes activating receptor–ligand interactions, amber the metabolic/TCR arm. The interactions summarise published mechanisms and constitute a hypothesis-generating framework requiring clinical validation. BTN, butyrophilin; DNAM-1, DNAX accessory molecule 1; MHC, major histocompatibility complex; MICA/B, MHC class I-related chain A/B; NKG2D, natural killer group 2 member D; TCR, T-cell receptor; TRAIL, TNF-related apoptosis-inducing ligand.

The mechanistic case for deploying γδ T cells against glioblastoma rests on the convergence of several recognition pathways onto ligands that glioma cells reliably express, together with the capacity of γδ cells to act on the stem-like populations that drive recurrence (Table 2, Figure 1).

### 4.1. NKG2D, DNAM-1 and Stress-Induced Ligands

Malignant transformation, genotoxic stress and the DNA-damage response up-regulate the NKG2D ligands MICA, MICB and the UL16-binding proteins, and these ligands are detectable on glioma cell lines and primary tumours [44,51]. Human γδ T cells lyse malignant glioma lines in a manner dependent on both NKG2D and the TCR, and this killing can be modulated pharmacologically: inhibition of the ADAM10 and ADAM17 sheddases, which cleave surface MICA/B, increases ligand density and enhances γδ-mediated lysis, whereas temozolomide exposure alters ligand expression in context-dependent ways [52]. Crucially, NKG2D ligands are expressed not only on bulk tumour but also on glioma stem-like cells, both in situ and in vitro, identifying a recognition route to the very cells that resist conventional therapy [53]. The DNAM-1 axis provides a parallel, non-redundant pathway through CD155 and CD112 [45], ligands whose relevance to γδ effector function has been demonstrated in other malignancies [46]. The redundancy of these activating inputs is strategically important, because it makes recognition robust to the loss of any single ligand.

It is worth pausing on why this redundancy matters more in glioblastoma than in many other settings. The tumour’s capacity to interconvert between cellular states and to regenerate from a stem-like reservoir means that the antigenic and ligand landscape is not static but actively remodelled under therapeutic pressure [18,20]. A recognition system keyed to a single determinant is therefore playing against a moving target, whereas an effector reading several stress-associated cues simultaneously is more likely to retain a foothold as the tumour evolves. The argument has a clear boundary, however: NKG2D ligands are themselves subject to transcriptional downregulation and proteolytic shedding [51], so redundancy reduces but does not abolish the risk of escape, and the durability of γδ recognition under sustained selective pressure in vivo has not been established.

### 4.2. Phosphoantigen Sensing and Metabolic Cues

Layered onto innate-receptor recognition is the phosphoantigen-sensing capacity of Vγ9Vδ2 cells. Gliomas, like other tumours with a dysregulated mevalonate pathway, can accumulate the endogenous phosphoantigen that engages the Vγ9Vδ2 TCR through the butyrophilin machinery [32,33,36], and aminobisphosphonate pretreatment can amplify this signal [38]. Because this recognition reports on a metabolic state rather than a lineage antigen, it is intrinsically resistant to the antigen-escape mechanisms that defeated EGFRvIII-directed approaches—an argument that is biologically coherent but, it must be stressed, has not been validated against clinical relapse in glioblastoma.

### 4.3. Cytokine Output, Cross-Talk with Adaptive Immunity, and Activity Against Stem-like Cells

γδ T cells are more than serial killers. Their secretion of interferon-γ can reprogramme the myeloid compartment and enhance antigen presentation, and their capacity to act as antigen-presenting cells positions them to prime αβ T-cell responses, in principle converting a transient cytotoxic burst into a broader and more durable response [13,14]. This bridging function is mechanistically appealing in a tumour whose endogenous T-cell response is exhausted and sequestered [29,30], although direct evidence that infused γδ cells reconstitute productive adaptive immunity in glioblastoma is lacking. The most therapeutically significant attribute may be the documented activity of γδ cells against cancer stem-like cells [48], combined with the demonstration that glioma stem-like cells display NKG2D ligands [53]: together these observations suggest that γδ cells can target the radioresistant, recurrence-driving fraction that conventional cytotoxic therapy spares [21]. The strength of this rationale should not, however, be mistaken for proof of clinical benefit.

## 5. Preclinical Evidence of γδ T Cells in Glioblastoma

The experimental literature on γδ T cells in glioblastoma is now nearly two decades old, and a critical reading reveals both genuine reproducible signals and persistent methodological limits (Table 3).

### 5.1. In Vitro Studies

The foundational observations were made with patient-derived and ex vivo expanded γδ cells. Bryant and colleagues characterised γδ T cells from glioblastoma patients and showed that expanded/activated cells lysed glioma lines and primary cultures, establishing the basic feasibility of the approach [54,55]. Mechanistic dissection by Chitadze and colleagues subsequently attributed glioma killing to combined NKG2D and TCR engagement and demonstrated that the lytic interaction could be tuned by sheddase inhibition and by temozolomide [52], while Flüh and colleagues extended the target range by documenting NKG2D-ligand expression on glioma stem-like cells [53]. More recent work has exploited the DNA-damage response therapeutically, showing that temozolomide combined with a poly(ADP-ribose) polymerase inhibitor up-regulates the NKG2D ligand ULBP1 and augments γδ cytotoxicity against glioblastoma [56]. These studies are mutually reinforcing, but they share the limitations of in vitro work: reliance on cell lines and short-term cytotoxicity readouts, effector-rich conditions that do not reproduce the suppressive tumour milieu, and donor-to-donor variability that is often underreported.

### 5.2. Animal Studies

In vivo evidence is more demanding and correspondingly more informative. Stereotaxic administration of allogeneic human Vγ9Vδ2 cells controlled the growth of human glioblastoma xenografts in the brains of immunodeficient mice, providing proof of principle for locoregional delivery and for an allogeneic product [57]. Studies in immunocompetent murine glioma models have charted the dynamics of circulating γδ activity and underscored the influence of the host environment [58], an important counterweight to xenograft work that lacks an intact immune system. The most translationally developed line of investigation is the ‘drug-resistant immunotherapy’ concept, in which γδ cells are rendered temozolomide-resistant by enforced expression of O6-methylguanine-DNA methyltransferase so that they can be administered concurrently with chemotherapy; this strategy killed glioblastoma lines during a chemotherapy challenge and improved control of primary high-grade gliomas in combined regimens [59,60]. The convergent message from chemo-sensitisation studies is that cytotoxic therapy and γδ recognition can be made to cooperate rather than conflict [38].

A caveat that pervades this animal literature, and that a careful reader should keep in view, is the limited fidelity of murine systems to human γδ biology. The phosphoantigen-sensing Vγ9Vδ2 subset that dominates human blood has no direct murine equivalent, so xenograft studies using human γδ cells lack a syngeneic context while immunocompetent murine models necessarily rely on a different γδ repertoire [12,58]. Neither system reproduces the human butyrophilin machinery, the human NKG2D-ligand repertoire and the human tumour microenvironment simultaneously. This species gap does not invalidate the preclinical signal, but it does mean that quantitative parameters—effective dose, persistence, the magnitude of chemo-sensitisation—cannot be read directly from mouse to patient, and it places a premium on early-phase human correlative data over further model refinement.

### 5.3. Strengths, Weaknesses and Remaining Uncertainties

Viewed as a whole, the preclinical corpus has real strengths: the anti-glioma activity of γδ cells has been reproduced across laboratories, models and γδ sources; the mechanisms invoked are concrete and measurable; and the field has progressed beyond simple cytotoxicity to address the practical problem of combining cells with chemotherapy. The weaknesses are equally clear and must temper enthusiasm. Much of the in vivo efficacy data derives from immunodeficient xenografts that cannot model the suppressive microenvironment, regulatory T cells and myeloid cells that dominate human disease [22,23]; durable survival benefit, as opposed to transient tumour-growth delay, is inconsistently demonstrated; and the trafficking and persistence of infused γδ cells within brain tumours remain poorly quantified. Correlative human data are limited to observations that γδ infiltration carries favourable prognostic associations in pan-cancer analyses [61,62] and that a distinctive Vγ9Vδ2 population preferentially infiltrates glioblastoma [63]—associations that are hypothesis-generating rather than causal. The honest conclusion is that γδ cells show consistent and mechanistically credible anti-glioma activity in models, but that efficacy in patients remains unproven.

A further, often unstated, limitation concerns the internal consistency of the field. The conceptual case for γδ effectors against high-grade glioma was articulated more than fifteen years ago [64], yet the intervening literature has tended to re-demonstrate cytotoxicity in new models rather than to resolve the questions that determine clinical utility: the absolute number of effectors required, the depth and duration of tumour control achievable, and the conditions under which γδ activity is sustained rather than transient. Heterogeneity in expansion protocols, effector-to-target ratios and readout assays makes quantitative comparison across studies difficult, and negative or equivocal results are likely underreported. None of this negates the reproducible positive signal, but it does mean that the field’s apparent coherence partly reflects methodological convergence rather than independent confirmation of clinically meaningful efficacy—an important distinction when weighing whether to advance the approach into larger trials.

**Table 3:** Representative preclinical and early-phase clinical studies of γδ T cells in glioblastoma. Selected for illustration; not an exhaustive list. Clinical entries are labelled by evidence level: INB-200 has now been reported as a peer-reviewed phase 1 study, whereas INB-400 remains at conference-abstract level.

Study	Model/Setting	γδ Source	Principal Finding	Key Limitation
Bryant 2009/2011 [54,55]	Glioma lines, primary cultures, xenograft	Patient-derived, expanded	Expanded γδ cells lyse glioma; feasibility established	Cell-line dependence; short-term assays
Lamb 2013 [59]	Glioblastoma lines + chemotherapy	MGMT-modified (drug-resistant)	γδ kill during TMZ challenge	In vitro; engineered resistance
Beck 2015 [58]	Immunocompetent murine glioma	Endogenous/adoptive	Host environment shapes γδ activity	Murine γδ biology differs from human
Chitadze 2016 [52]	Malignant glioma lines	Expanded human γδ	NKG2D + TCR killing; sheddase/TMZ modulation	In vitro mechanism
Jarry 2016 [57]	Intracranial human GBM xenograft	Allogeneic Vγ9Vδ2	Stereotaxic γδ control tumour growth	Immunodeficient host
Flüh 2018 [53]	Glioma stem-like cells	—	NKG2D ligands on GSCs in situ/in vitro	Expression study; no efficacy endpoint
Lamb 2021 [60]	Primary high-grade glioma	MGMT-modified γδ + TMZ	Combined regimen effective	Model-level efficacy; not survival in patients
Jones 2024 [56]	GBM models	Expanded human γδ	TMZ + PARP inhibitor raise ULBP1, boost killing	In vitro/preclinical
IN8bio INB-200/400 [65,66]	Phase 1/1b (newly diagnosed/recurrent GBM)	Autologous or allogeneic DRI γδ + TMZ	Early feasibility/safety signals reported	Conference abstracts; INB-200 has since been reported in full [67]
Park 2021 [25]	Immunocompetent brain tumour models	Endogenous and adoptive γδ	Tumour hypoxia represses NKG2D through protein kinase A; relieving hypoxia restores γδ function	Mechanism shown in models, not in patients
Nabors 2026 [67]	Phase 1, newly diagnosed GBM (13 treated)	Autologous MGMT-modified (DRI) γδ, intracavitary, with TMZ	No dose-limiting toxicity, CRS or ICANS; mPFS 9.9 months (16.1 months with repeated dosing); mOS 15.6 months	Single-arm, small; not powered for efficacy; no comparator

DRI, drug-resistant immunotherapy; GBM, glioblastoma; GSC, glioma stem-like cell; MGMT, O6-methylguanine-DNA methyltransferase; PARP, poly(ADP-ribose) polymerase; TCR, T-cell receptor; TMZ, temozolomide; CRS, cytokine release syndrome; ICANS, immune effector cell-associated neurotoxicity syndrome; mOS, median overall survival; mPFS, median progression-free survival; NKG2D, natural killer group 2 member D.

## 6. Opportunities for γδ T Cell-Based Immunotherapy in Glioblastoma

**Figure 2:**
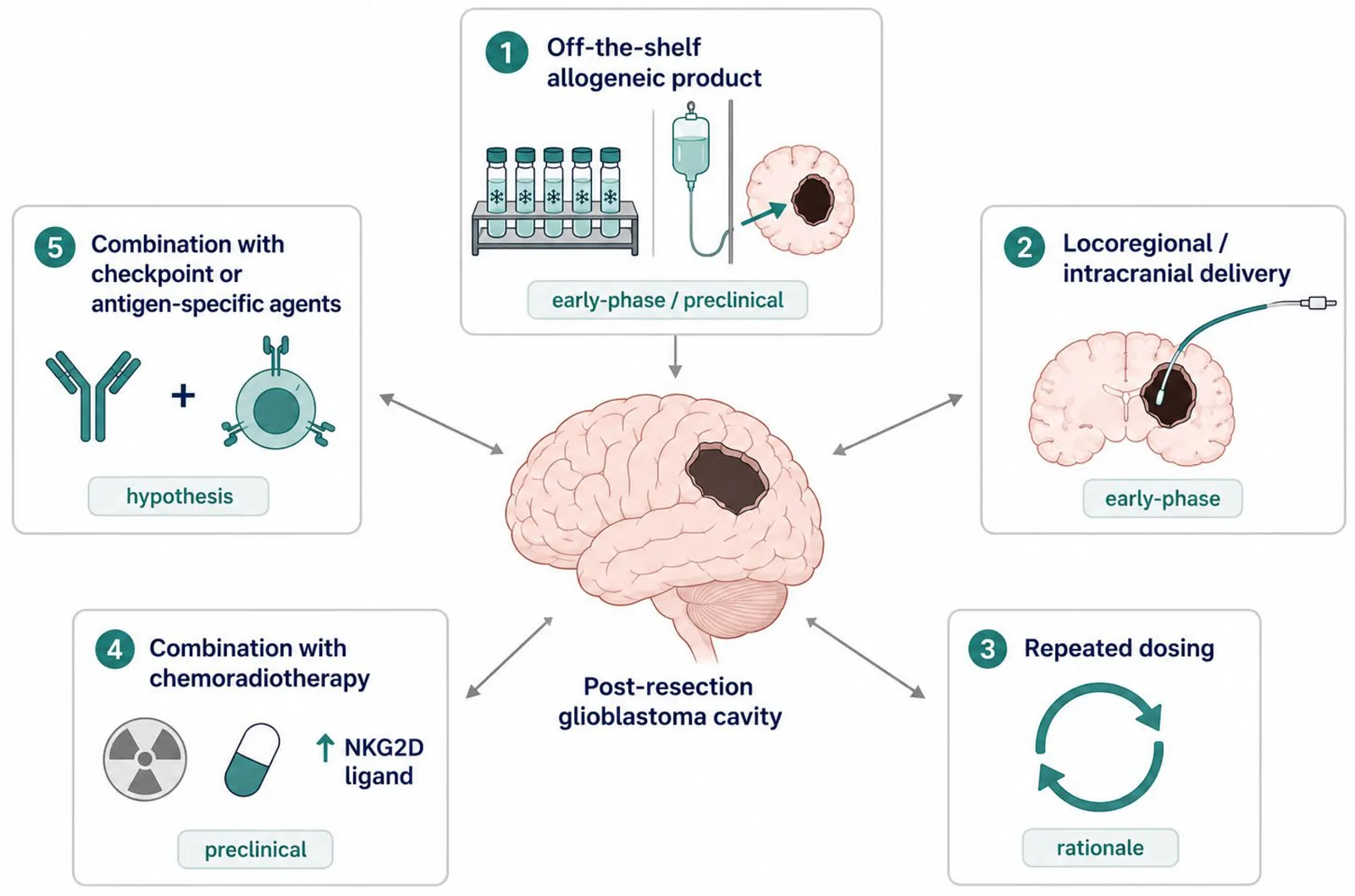
Potential clinical applications of γδ T cells in glioblastoma. Conceptual schematic, not to scale. Five candidate applications are arranged around a post-resection glioblastoma cavity, each tagged by the maturity of its supporting evidence. (1) An allogeneic, off-the-shelf γδ product—banked from qualified healthy donors and engageable across HLA-disparate recipients with minimal graft-versus-host risk—delivered intravenously or intracavitarily (early-phase/preclinical). (2) Locoregional or intracranial delivery via an indwelling catheter that partly bypasses the blood–brain barrier (early-phase). (3) Repeated dosing to offset the short persistence of unmodified γδ cells, made practical by an inventory product (rationale). (4) Combination with chemoradiotherapy, exploiting genotoxic up-regulation of NKG2D ligands and the drug-resistant-immunotherapy design that permits γδ cells to function during temozolomide exposure (preclinical). (5) Combination with immune checkpoint inhibitors or antigen-specific agents (bispecific antibodies or chimeric antigen receptors) to hedge against antigen escape (hypothesis). The colour scheme matches Figure 1; none of the depicted applications constitutes an established treatment. HLA, human leukocyte antigen; NKG2D, natural killer group 2 member D.

Several features of γδ biology align with the specific obstacles glioblastoma presents, and these alignments define the principal opportunities for the field (Figure 2).

### 6.1. Allogeneic, Off-the-Shelf Products and Repeated Dosing

Because γδ recognition is MHC-independent and γδ cells are not expected to mediate graft-versus-host disease, healthy-donor cells can in principle be banked, qualified and administered across HLA-disparate recipients—the off-the-shelf model that allogeneic cell-therapy developers have pursued to overcome the cost, delay and failure rate of patient-specific manufacturing [15,68]. The clinical safety of allogeneic and haploidentical γδ transfer has been reported in early-phase studies in other settings [37,69,70], and an allogeneic stereotaxic approach was effective in a glioblastoma xenograft model [57]. For a tumour that recurs predictably, the practical corollary—an inventory product available for repeated dosing without re-manufacturing—is particularly valuable, since the limited persistence of unmodified γδ cells may otherwise blunt single-dose strategies.

### 6.2. Locoregional and Intracranial Delivery

The barrier-access problem that constrains systemic therapy is partly circumvented by delivering cells directly to the tumour or resection cavity. The principle is established for CAR-T cells in glioblastoma, where locoregional administration was associated with biological activity and acceptable tolerability [71,72], and it extends naturally to γδ products: stereotaxic delivery of allogeneic Vγ9Vδ2 cells controlled intracranial xenografts [57], and intracranial administration of gene-modified γδ cells is the explicit design of the most advanced clinical programme [73]. Locoregional delivery also concentrates effectors at the infiltrative margin behind the intact blood–brain barrier [28], though it introduces its own surgical and logistical demands.

Locoregional delivery is nonetheless not a panacea, and its practical demands deserve explicit acknowledgement. Repeated intracavitary or intraventricular dosing requires an indwelling device and the associated infection and procedural risks; the distribution of cells from the instillation site into infiltrated parenchyma is uneven and incompletely characterised [57,73]; and the approach concentrates effort at the resection cavity while the most clinically dangerous disease is often the diffuse, non-resectable infiltrate. These considerations do not negate the rationale—delivering cells to the tumour bed is more efficient than relying on systemic trafficking across a heterogeneously intact barrier [28]—but they temper the expectation that route of administration alone will overcome the access problem, and they argue for combining locoregional delivery with strategies that promote intratumoural migration and persistence.

### 6.3. Combination with Chemoradiotherapy

The standard of care may be not merely compatible with but synergistic toward γδ therapy. Genotoxic stress from radiation and temozolomide up-regulates NKG2D ligands and can sensitise tumour cells to γδ recognition [38,56], and the drug-resistant-immunotherapy strategy was conceived specifically to allow γδ cells to function during, rather than after, chemotherapy by engineering temozolomide resistance into the effector [59,60]. This represents a rational integration with existing treatment rather than a competing modality, an advantage given the entrenched role of chemoradiotherapy. The optimal sequencing and dosing, however, remain to be defined empirically. This section presents the interaction as an opportunity; Section 7.3 sets out the countervailing evidence that genotoxic therapy also recruits and polarises endogenous γδ cells in ways that may favour the tumour, and the two should be read together.

The interaction with chemotherapy is more nuanced than simple synergy, and the tension deserves explicit statement. Temozolomide is itself lymphodepleting, and the standard regimen contributes to the systemic T-cell deficits that already characterise the disease [4,30]; an unprotected cellular product administered during chemotherapy would be exposed to the same cytotoxic pressure as the tumour. The drug-resistant-immunotherapy strategy addresses exactly this conflict by engineering temozolomide resistance into the effector so that chemotherapy and cell therapy can be co-administered without mutual antagonism [59,60], but this solution adds genetic modification—and therefore manufacturing and regulatory complexity—to what is otherwise an unmodified product. The trade-off between the simplicity of an unengineered γδ cell and the schedule flexibility conferred by drug resistance is a genuine design decision rather than a settled question, and the right answer may differ by clinical setting.

### 6.4. Combination with Checkpoint Blockade and Antibody-Based Redirection

Although checkpoint blockade has underperformed as monotherapy in glioblastoma [7], neoadjuvant PD-1 blockade can elicit measurable intratumoural and systemic immune responses [74,75], providing a rationale for combining checkpoint modulation with an effector population that is itself susceptible to exhaustion under chronic stimulation [76]. Separately, the antigen-agnostic breadth of γδ recognition could complement antigen-specific redirection: bispecific antibodies and engineered cells targeting glioma antigens such as EGFRvIII have entered the clinic [77,78], and pairing a defined-antigen agent with an effector that also reads stress ligands is a conceptually attractive way to hedge against antigen escape. These combinations are at present hypotheses; none has been validated in glioblastoma, and each adds toxicity and trial-design complexity that must be weighed.

## 7. Challenges and Barriers

**Figure 3:**
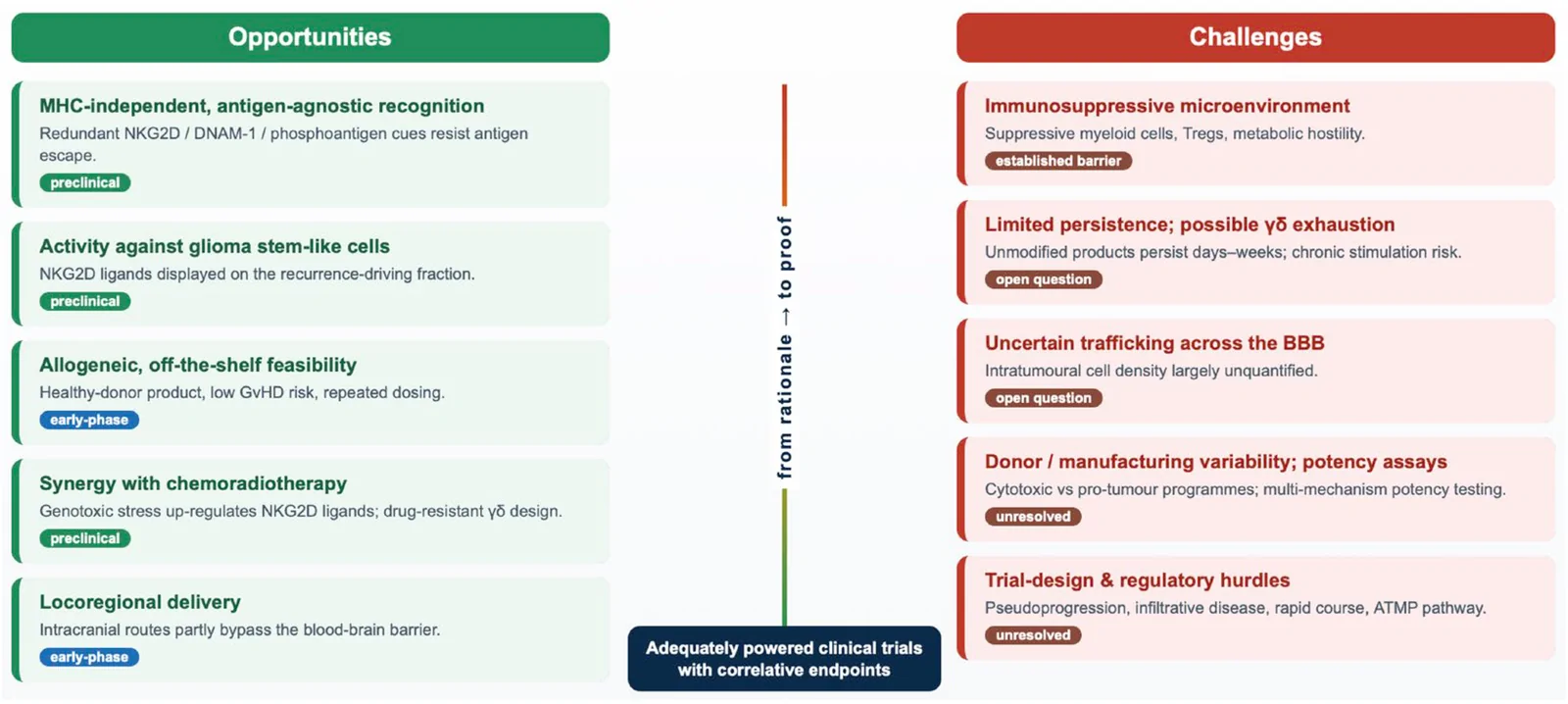
Challenges and opportunities of γδ T-cell therapy in glioblastoma. Conceptual schematic. A balanced juxtaposition of the mechanistic and logistical advantages of γδ T cells (left, green) against the biological and translational barriers that remain unresolved (right, red). Opportunities span MHC-independent, antigen-agnostic recognition; activity against glioma stem-like cells; allogeneic off-the-shelf feasibility with low graft-versus-host disease (GvHD) risk; synergy with chemoradiotherapy through NKG2D-ligand induction; and locoregional delivery. Challenges span the immunosuppressive microenvironment; limited persistence and possible γδ exhaustion; uncertain trafficking across the blood–brain barrier (BBB); donor and manufacturing variability with attendant potency-assay difficulty; and trial-design and regulatory hurdles for an advanced therapy medicinal product (ATMP). Each item is tagged by evidence status (preclinical, early-phase, or open question). The central ‘rationale → proof’ axis emphasises that the gap between a coherent biological rationale and demonstrated clinical benefit can be closed only by adequately powered trials incorporating correlative endpoints.

The same disease features and product characteristics that create opportunities also impose formidable barriers, and a balanced account must give them equal weight (Figure 3).

### 7.1. Tumour Immunosuppression and Effector Persistence

γδ cells delivered into a glioblastoma must function within the suppressive milieu described above, with its regulatory T cells, suppressive myeloid cells and metabolic constraints [22,23,24]. γδ cells are not immune to this environment: intratumoural γδ populations can acquire exhaustion features, although some retain function [76], and the same chronic stimulation that exhausts αβ cells is likely to affect γδ cells. Compounding this, unmodified allogeneic γδ products typically persist for days to a few weeks [37], a window that may be too short to achieve durable control of an infiltrative tumour and that argues for cytokine support or repeated dosing whose optimal form is undefined.

Persistence is further constrained by an issue intrinsic to the off-the-shelf model that is easy to overlook amid its logistical appeal: host rejection of allogeneic cells. The same MHC independence that spares recipients from graft-versus-host disease does not protect the infused product from the patient’s own residual immune system, and host-versus-graft alloreactivity is a recognised limit on the persistence and repeat-dosing utility of allogeneic cellular therapies generally [68]. For engineered allogeneic αβ products, this has motivated gene editing to evade rejection; for an unmodified γδ product the trade-off is starker, because the very simplicity that is its regulatory advantage leaves it without such protection. How quickly allogeneic γδ cells are cleared in glioblastoma patients—many of whom are lymphodepleted by prior therapy [30], which could paradoxically prolong persistence—is unknown and is an important variable for the repeated-dosing strategy on which the off-the-shelf model depends.

### 7.2. Exhaustion Under Chronic Stimulation and Metabolic Fitness

A recurring assumption in the γδ literature is that innate-like effectors are less vulnerable than conventional T cells to the dysfunction imposed by chronic antigen exposure. The evidence supports a more qualified statement. Intratumoural γδ populations do acquire inhibitory-receptor expression and functional impairment, but the phenotype is not a copy of αβ exhaustion. In human kidney cancer, Vδ2-negative γδ cells bearing an exhausted surface phenotype nevertheless retained effector function on restimulation [76]; single-cell profiling of triple-negative breast cancer found tumour-infiltrating Vγ9Vδ2 cells to be PD-1-low and TOX-low, with muted T-cell-receptor signalling and a skew towards early effector-memory differentiation, accompanied—tellingly—by little response to PD-1 blockade and by restoration of cytotoxic type 1 function when butyrophilin 3A was engaged instead [79]; and in multiple myeloma, bone-marrow Vγ9Vδ2 cells accumulate checkpoint expression and lose function as disease advances [80]. Read together, these studies suggest that the dominant lesion in tumour-associated Vγ9Vδ2 cells is hyporesponsiveness at the level of receptor triggering rather than the terminal, TOX-driven exhaustion programme of chronically stimulated CD8 cells. The distinction has practical consequences, because it predicts that checkpoint blockade alone will achieve less for γδ cells than agonism of their activating machinery. Whether the same holds in glioblastoma, where the exhaustion of resident αβ cells is unusually severe [29], has not been examined: no study has profiled the differentiation or exhaustion state of γδ cells recovered from human glioblastoma after infusion, and that is a conspicuous gap.

The metabolic question is more tractable, and the answer is less comfortable than the phrase ‘innate-like metabolic flexibility’ implies. Expanded Vγ9Vδ2 cells rest in a quiescent state that draws on both glycolysis and oxidative phosphorylation, but they up-regulate both arms upon receptor triggering and depend specifically on glycolysis and the pentose phosphate pathway for proliferation; their killing of tumour targets is blunted by glycolytic inhibition and by glucose withdrawal while remaining largely intact when oxidative phosphorylation is inhibited [81]. Effector function is therefore glucose-dependent in precisely the way that a nutrient-depleted, myeloid-rich tumour bed penalises [22,24]. Oxygen imposes a second and, for brain tumours, a specific constraint: brain tumour cells consume oxygen at unusually high rates, and the resulting hypoxia acts on γδ cells through transcriptional activation of protein kinase A to repress NKG2D—an effect that spared conventional T cells in the same models and that was reversed when tumour hypoxia was relieved [25]. This is the most direct available answer to the question of whether γδ metabolic flexibility survives the conditions of glioblastoma: in a brain tumour model, it did not, and the principal recognition receptor invoked throughout this review was the casualty. Murine work adds that interferon-γ-producing and interleukin-17-producing γδ subsets rest on distinct metabolic programmes [82], so metabolic pressure is likely to shift the functional balance of a product rather than merely to dampen it uniformly.

Two practical conclusions follow. First, potency and comparability testing performed under nutrient-replete normoxia—the standard for release assays—will systematically overstate what a product does in situ; characterisation under low oxygen tension, low glucose and physiological lactate is a feasible addition to a preclinical package and would make in vitro cytotoxicity a more honest predictor of behaviour in the tumour. Second, the lesions identified so far are addressable rather than fixed: expansion in interleukin-2 together with interleukin-15 yields Vγ9Vδ2 cells with higher perforin, granzyme B and granulysin content, higher T-bet expression and greater cytotoxicity that is retained under hypoxia [83]; relief of tumour hypoxia restored NKG2D expression and γδ function in vivo [25]; and butyrophilin agonism restored cytotoxic function in cells that checkpoint blockade did not reach [79]. These are the levers presently available, and Section 9.2 considers them alongside the engineering options.

### 7.3. γδ T Cells and Genotoxic Therapy: A Double-Edged Axis

Section 6.3 presented the interaction between γδ cells and the genotoxic standard of care as an opportunity, and at the level of the tumour cell, it is one. A recent line of work requires that the account be balanced by its opposite. In mouse tumour models, radiotherapy substantially increased γδ infiltration, and the influx contributed to radioresistance rather than to tumour control: depletion of γδ cells improved radiosensitivity, and single-cell profiling identified the post-radiotherapy γδ compartment as Zbtb16-, Il23r- and Il17a-expressing and as the principal source of interleukin-17A, which recruited myeloid-derived suppressor cells and suppressed T-cell activation. Mechanistically, irradiated tumour cells shed microparticles containing double-stranded DNA that activated cGAS-STING and NF-κB signalling in macrophages, up-regulating the chemokine CCL20 and thereby drawing γδ cells in; targeting γδ cells or interleukin-17A increased radiosensitivity and improved the effect of radiotherapy combined with PD-1 blockade [84]. The accompanying commentary frames the finding as a caution for radioimmunotherapy design rather than as an argument against γδ-directed approaches [85].

Two qualifications are essential before this is imported into glioblastoma. The models were not intracranial, and the responding cells were murine γδ subsets with no direct counterpart to the human Vγ9Vδ2 population that dominates clinical products [12,58]; the observation therefore describes the endogenous γδ compartment reacting to irradiation, not the behaviour of an ex vivo expanded, phenotypically selected, intracranially delivered product. With that said, the elements of the pathway are conspicuously present in the treated glioblastoma bed. Irradiation generates cytosolic DNA and damage-associated signals in a compartment where microglia and macrophages are the dominant immune population [22,23], and microglia activated through Toll-like receptors are themselves capable of polarising γδ cells towards interleukin-17 production by way of interleukin-1β and interleukin-23 [26]; interleukin-17-producing γδ cells are the pro-tumourigenic arm of the lineage in other settings [49,50]. The field has generally treated the induction of NKG2D ligands by radiotherapy as an unmixed benefit. The more accurate statement is that genotoxic therapy remodels the effector compartment as well as the target, and that the net effect depends on which γδ programme prevails locally.

For product development, this converts an abstract concern into concrete requirements. A cytotoxic, interferon-γ-biased phenotype should be an explicit release attribute rather than an assumed property of the expansion protocol, with interleukin-17 production measured rather than presumed absent [50]; correlative sampling in trials that combine cells with radiotherapy should quantify the endogenous γδ and interleukin-17 axis alongside the infused product, since the two may move in opposite directions; and the interleukin-23/interleukin-17 axis becomes a rational candidate for pharmacological control within combination regimens. It also sharpens a scheduling question that the field has largely left to convenience: whether cells are best delivered within the window in which stress ligands are induced, or after any recruitment-and-suppression wave has resolved, is an empirical question that the present data cannot settle.

### 7.4. Corticosteroids and Concomitant Medication

An obstacle peculiar to glioblastoma, which immunotherapy trials in this disease can neither ignore nor easily design around, is the near-ubiquitous use of corticosteroids. Dexamethasone is administered to most patients at some point for vasogenic oedema, and its immunological effects are neither subtle nor confined to the tumour: it reduces lymphocyte numbers by promoting apoptosis, impairs the functional capacity of the cells that remain, and depresses myeloid and natural killer populations as well [86,87]. In immunocompetent glioblastoma models, concurrent dexamethasone reduced survival after PD-1 blockade in a dose-dependent manner and abrogated the benefit of PD-1 blockade given with or without radiotherapy; in a retrospective series of 181 patients with isocitrate dehydrogenase-wild-type glioblastoma treated with PD-(L)1 blockade, baseline dexamethasone emerged as the strongest independent predictor of poor survival on multivariable adjustment [87]. Corticosteroid exposure during chemoradiotherapy is an independent risk factor for severe lymphopenia, which is itself associated with worse outcome [88,89]. The analogous experience in cellular therapy outside the central nervous system points the same way, corticosteroid administration having been associated with inferior outcomes after CD19-directed CAR T-cell therapy in large B-cell lymphoma [90].

No study has measured the effect of dexamethasone on human γδ T cells in a therapeutic context, and it would be wrong to assert a γδ-specific sensitivity that has not been demonstrated. The general mechanisms are not, however, ones from which γδ cells would obviously be exempt: they are lymphocytes that depend on interleukin-2 and interleukin-15 signalling, on cytokine-driven proliferation and on interferon-γ output for the functions described throughout this review. A locoregionally delivered product may be partly insulated from systemic exposure, but dexamethasone reaches the tumour bed freely, and steroid effects on the myeloid compartment will alter the environment the cells enter irrespective of route. Three practical implications follow: corticosteroid dose should be recorded as a prespecified covariate rather than as a background variable; steroid-sparing management of oedema should be pursued where clinically defensible; and eligibility criteria should state a maximum dexamethasone dose at the time of cell administration, as is now usual in checkpoint and CAR T-cell studies in this disease. Measuring γδ function directly in patients on and off corticosteroids would be a straightforward and worthwhile correlative endpoint.

### 7.5. Trafficking and the Blood–Brain Barrier

For systemically administered cells, reaching the infiltrative tumour behind a heterogeneously intact barrier is a fundamental obstacle [28], and the biodistribution of infused γδ cells in brain tumours is poorly characterised. Locoregional delivery mitigates but does not eliminate the problem, because cells must still migrate from the resection cavity into infiltrated parenchyma [57,73]. Quantitative trafficking data—how many cells reach the tumour, where, and for how long—are largely absent and represent a first-order gap for the field.

Part of the difficulty is methodological. Tracking the fate of infused cells in the brain demands labelling and imaging approaches whose sensitivity and quantitative accuracy are limited, and most preclinical glioma work has relied on endpoint histology or on xenograft systems that do not reproduce human trafficking [57,58]. Without serial, quantitative readouts of where γδ cells go and how long they remain, it is impossible to distinguish failure of trafficking from failure of function as the cause of a disappointing result—a distinction with direct therapeutic consequences, since the two demand opposite remedies. Incorporating cell-tracking and pharmacodynamic biomarkers into early-phase studies is therefore not an academic refinement but a prerequisite for rational iteration, and its current absence is among the clearest reasons that the field’s in vivo claims remain provisional.

### 7.6. Manufacturing, Donor Heterogeneity and Potency Assays

Translating γδ therapy at scale exposes manufacturing challenges. Although expansion protocols using zoledronate and interleukin-2 are well established [39], the functional output varies with donor and method, and the coexistence of cytotoxic and pro-tumour γδ programmes [49,50] makes donor selection and phenotype control consequential rather than incidental. As advanced therapy medicinal products, γδ cells must satisfy demanding requirements for characterisation, comparability and potency; potency-assay development—linking a measurable bioassay to the clinical mechanism of action—is a recognised and recurrent obstacle for cell therapies [91,92], and for a product with several non-redundant killing mechanisms, the assay must capture more than one axis of function.

### 7.7. Regulatory and Clinical-Trial-Design Challenges

Beyond manufacturing, the regulatory pathway for an allogeneic cell therapy is exacting [68,92], and glioblastoma poses particular trial-design difficulties: response assessment is confounded by treatment-related imaging changes, the infiltrative disease defies conventional measurement, and the rapid clinical course compresses the window for adaptive cell therapy. The experience of CAR-T development in glioblastoma, where biological activity has not yet translated into survival benefit, is a cautionary precedent [93,94]. Designing trials that can detect a true effect—through appropriate endpoints, biomarker integration and realistic comparators—is itself a substantial scientific challenge.

## 8. Comparison with Other Cell-Based Immunotherapies

γδ T cells are one of several cellular platforms under investigation for glioblastoma, and their place is best understood by comparison rather than in isolation (Table 4). Engineered αβ CAR-T cells offer potent, defined-antigen cytotoxicity and have produced the most striking individual responses in the disease—including a widely cited regression after IL-13Rα2-directed therapy [71] and subsequent locoregional trials [72]—but they depend on a target antigen and are therefore vulnerable to the antigen heterogeneity and escape that characterise glioblastoma [8,9], and in their autologous form they inherit the manufacturing burden and the host-lymphopenia problem of the disease [30]. TCR-engineered αβ cells broaden the targetable antigen space to intracellular proteins but remain MHC-restricted and have been developed chiefly outside the CNS [95,96]. Tumour-infiltrating lymphocyte therapy, transformative in melanoma [97,98], is constrained in glioblastoma by the exhausted and sparse intratumoural T-cell pool [29]. A notable recent counterpoint is the activity of GD2-directed CAR-T cells in H3K27M-mutated diffuse midline glioma, which produced radiographic and clinical responses and demonstrated that engineered cells can mediate meaningful effects in the CNS when antigen expression is high and homogeneous [99]—precisely the conditions that glioblastoma, with its antigenic heterogeneity, rarely satisfies.

Natural killer (NK) cells share with γδ cells an MHC-unrestricted, stress-ligand-based recognition and a favourable allogeneic safety profile, and CAR-NK products have shown activity without major toxicity in haematological disease [100], with NK-based strategies under active study in glioma [101]; however, NK cells lack a CD3-based TCR and so cannot be redirected by CD3-engaging bispecifics, and they too are susceptible to microenvironmental suppression. Within this landscape, γδ cells occupy a distinctive position: they combine NK-like innate recognition with a rearranged, CD3-coupled TCR [41], recognise tumours without MHC restriction, pose minimal graft-versus-host risk in the allogeneic setting, and can act on stem-like cells [53]. This combination, rather than superiority on any single axis, is what motivates their continued development; the comparison is one of complementary trade-offs, and γδ cells are best regarded as an addition to, not a replacement for, the cellular armamentarium [102,103].

Framing the comparison this way also clarifies where γδ cells are most likely to earn a place. They are unlikely to outperform a well-matched CAR-T product against a homogeneously expressed antigen, and they are not a solution to the delivery problem that constrains every cellular therapy in the brain. Their distinctive value lies instead in settings that defeat antigen-specific approaches: heterogeneous or antigen-low tumours, post-relapse disease in which a target has been lost, and the stem-like fraction that displays stress ligands but few lineage antigens [53]. This suggests that the most informative early trials may not pit γδ cells against established platforms head-to-head, but rather position them where the alternatives are weakest—as a complementary effector layered onto standard therapy, or as a rational partner for antigen-specific agents whose principal failure mode is escape [8,9]. Whether that theoretical niche translates into measurable benefit is, once again, a question for trials rather than for argument.

## 9. Clinical Translation and Future Perspectives

### 9.1. Clinical Status: From Conference Abstracts to the First Peer-Reviewed Report

Clinical translation of γδ T-cell therapy for glioblastoma is in its earliest stage, although the evidentiary situation changed materially during the preparation of this review. The most advanced effort is the drug-resistant-immunotherapy programme, in which gene-modified, temozolomide-resistant γδ cells are administered intracranially alongside maintenance chemotherapy; the autologous INB-200 study and the allogeneic/autologous INB-400 study first reported preliminary feasibility and safety in conference proceedings, with the strategy and design described in detail [65,66,73]; updated interim data from these programmes continued to be presented in abstract form [104] (Table 5). The phase 1 INB-200 experience has since been reported in full [67]. Of 23 patients enrolled, 13 received ex vivo expanded γδ T cells transduced with a methylguanine-DNA methyltransferase-expressing lentivector, delivered into the resection cavity through a Rickham catheter at 1 × 10^7^ cells per dose in escalating schedules of one, three or up to six doses, each given with a maintenance temozolomide cycle. No dose-limiting toxicity, cytokine release syndrome or immune effector cell-associated neurotoxicity syndrome was observed. At a median follow-up of 15.6 months, median progression-free survival was 8.0 months in the single-dose cohort, 9.9 months across all treated patients and 16.1 months in the repeat-dosing cohorts, with a median overall survival of 15.6 months for the treated population [67]. Beyond this programme, an allogeneic, gene-edited γδ product is in early-phase evaluation in recurrent glioblastoma in China, and an allogeneic CAR-γδ product includes glioblastoma in a solid-tumour expansion cohort, but neither has reported clinical data. It is important to be precise about the maturity of this evidence: apart from the INB-200 report, the clinical data exist principally as meeting abstracts and trial registrations rather than as peer-reviewed reports with mature survival outcomes, and no randomised comparison is available for any γδ product in this disease. Enthusiasm should therefore be calibrated to feasibility and safety signals, not efficacy.

This publication is a material advance in the quality of the evidence, and it should be read for what it can and cannot support. Its principal contributions are a peer-reviewed safety data set—repeated intracranial delivery of a gene-modified cellular product alongside chemotherapy without dose-limiting toxicity, cytokine release syndrome or neurotoxicity—and a demonstration that the manufacturing and delivery model is workable in routine outpatient practice. It is not evidence of a survival benefit. Thirteen treated patients distributed across three dose–schedule cohorts in a single-arm study cannot support a comparison with historical outcomes; the reported survival figures fall within the range described for contemporary chemoradiotherapy cohorts [3,4]; the longer progression-free survival in the repeat-dosing cohorts is subject to the guarantee-time bias that affects any comparison conditioned upon having received multiple doses; and the study was neither designed nor powered for efficacy. Comparable data for INB-400 remain at conference-abstract level, and the two other registered γδ programmes in glioblastoma have not reported clinical results (Table 5). The reading of the clinical evidence as a whole is therefore unchanged in direction and firmer in foundation: safety and feasibility can now be discussed with reference to a peer-reviewed report, whereas efficacy remains undemonstrated, and enthusiasm should be calibrated accordingly.

The contemporary clinical landscape for γδ therapy in glioblastoma is consequently sparse and concentrated in a small number of academic and company-sponsored programmes, with most registered studies in the phase 1 range and reported chiefly through trial registrations and meeting proceedings, the single-arm phase 1 INB-200 study being the only one so far reported as a peer-reviewed primary article [65,66,67,73]. This evidentiary immaturity is not a criticism of the investigators but a description of where the field stands, and it has practical implications for interpretation: safety can be inferred with reasonable confidence from early experience, whereas any claim of clinical benefit awaits controlled data. Trial design will be decisive. Glioblastoma studies are confounded by pseudoprogression and treatment-related imaging change, by an infiltrative disease that resists conventional measurement, and by a clinical course rapid enough to outpace adaptive manufacturing—obstacles that have repeatedly complicated the interpretation of cellular and immunotherapeutic trials in this disease [93,94]. Embedding correlative endpoints—quantitative measures of γδ trafficking and persistence, NKG2D-ligand and CD155 expression on tumour, and pharmacodynamic markers of effector activity—will be essential to convert feasibility studies into mechanistically informative ones, and to identify in advance the patients and tumour profiles most likely to benefit.

### 9.2. Engineering and Pharmacological Strategies to Sustain γδ Function

The barriers set out in Section 7 are, for the most part, not arguments against the platform but a specification for the next generation of products. Three families of intervention follow directly from the deficits documented above: cytokine support to counter short persistence and modest effector arming; shielding from the suppressive and metabolic signals of the tumour bed; and deliberate management of the DNA-damage and innate-sensing response that genotoxic therapy sets in motion. Table 6 summarises the candidates, the deficit each addresses, the evidence behind it and the cost it imposes. Two general cautions apply throughout. Much of the supporting evidence comes from αβ CAR T-cell engineering and has not been reproduced in γδ cells, so transfer is an assumption rather than a finding; and every modification converts an unmodified cellular product into a genetically modified one, with the manufacturing, comparability and regulatory burden discussed in Section 7.6 and Section 7.7 [91,92].

Cytokine support is the most immediately actionable. Unmodified γδ products persist for days to a few weeks [37], and interleukin-2, the mainstay of expansion protocols [39], supports proliferation more than it arms the cytotoxic programme. Supplementing expansion with interleukin-15 yields Vγ9Vδ2 cells with higher perforin, granzyme B and granulysin content, higher T-bet expression and greater cytotoxicity that is retained under hypoxia [83]—an ex vivo intervention that requires no genetic modification and is therefore available to any manufacturer. Where persistence rather than potency is limiting, cell-intrinsic cytokine armouring is the logical extension: a Vδ1 product engineered to secrete soluble interleukin-15 alongside a chimeric antigen receptor showed sustained antitumour activity preclinically [40], and CAR-modified Vγ9Vδ2 cells have been propagated for allogeneic use using a bisphosphonate prodrug [105]. The trade-off is that autocrine cytokine support raises the toxicity and manufacturing questions that have accompanied armoured CAR T cells generally, and no such construct has been evaluated intracranially.

Shielding addresses the suppressive milieu itself. Transforming growth factor-β is abundant in glioblastoma and inhibits cytotoxic lymphocytes; expression of a dominant-negative receptor, or equivalent disruption of the pathway, restores function in antigen-specific αβ T cells and is a natural candidate for γδ products [106]. The metabolic lesions of Section 7.2 suggest two further and less explored targets: relief of tumour hypoxia restored NKG2D expression and γδ function in a brain tumour model [25], and interruption of the adenosine axis is under active investigation in glioblastoma, where CD73 inhibition constrained tumour growth and reshaped the microglial compartment in mice [107]. Protection against host rejection is a distinct problem for allogeneic products, and the editing strategies developed for allogeneic αβ cells define the available options [68]. In each case the honest statement is the same: the deficit is documented in γδ cells, whereas the remedy has so far been demonstrated in another cell type.

The third family is specific to glioblastoma, because its standard of care is genotoxic and because Section 7.3 showed that genotoxicity cuts both ways. On the target side the pathway is well mapped: alkylator- and radiation-induced activation of the DNA-damage response up-regulates NKG2D ligands [44,51], an effect that can be amplified by combining temozolomide with a poly(ADP-ribose) polymerase inhibitor [56] or by blocking ADAM10- and ADAM17-mediated shedding of MICA and MICB [52]; NKG2D-directed effectors and subtherapeutic radiotherapy acted synergistically in immunocompetent glioma models, radiotherapy promoting migration to the tumour and enhancing effector function [108]. On the effector side the requirement is resistance to the alkylator itself, which the methylguanine-DNA methyltransferase-modified design supplies and which the INB-200 report shows to be compatible with concurrent temozolomide in patients [59,60,67]. The innate-sensing arm is where the field must be most careful. cGAS-STING signalling can be exploited: STING agonism reprogrammed the glioblastoma immune microenvironment and prolonged survival in preclinical models [109], and a STING agonist combined with radiotherapy produced long-term survival in one immunocompetent glioma model while transiently opening the blood–brain barrier—a delivery consideration of obvious relevance to a cellular product that must cross it [110]. Yet the same axis, acting through macrophage-derived CCL20, recruited interleukin-17-producing γδ cells and promoted radioresistance in non-CNS models [84]. Whether STING activation in the irradiated glioblastoma bed would help or hinder a γδ product is therefore genuinely unresolved, and the components that decide the answer—the local interleukin-23/interleukin-17 axis, CCL20-driven recruitment and the phenotype of the infused cells—are precisely those an engineering programme should be measuring and, where possible, controlling.

Finally, additivity cannot be inferred from the mechanism; it has to be built into the schedule. The available data support a defensible starting hypothesis rather than an established regimen: deliver cells within the window in which the DNA-damage response has raised stress-ligand density but before any suppressive recruitment wave matures; protect the product against the alkylator to which it will be exposed; arm it so that its cytotoxic programme survives hypoxia and glucose competition; and keep it phenotypically stable against interleukin-17 polarisation. Each element is independently testable in existing preclinical models and none requires a new platform. The value of stating them together is that it makes explicit what a combination trial is assuming, and what an unexpected result would mean.

### 9.3. Research Priorities

Looking ahead, several directions follow from the evidence rather than from speculation. Engineering approaches that confer chemotherapy resistance [59,60], arm γδ cells with chimeric antigen receptors [40], or provide cytokine support to extend persistence are logical responses to the documented limitations of unmodified products. Rational integration with the standard of care, exploiting genotoxic up-regulation of NKG2D ligands [38,56], is a near-term opportunity that does not require displacing existing therapy. Allogeneic, off-the-shelf manufacturing [68] would make repeated locoregional dosing practical if persistence remains short. The major unanswered questions are concrete: whether infused γδ cells reach and persist at the infiltrative tumour in sufficient numbers; whether their activity survives the suppressive microenvironment; which patients, tumours and ligand profiles predict benefit; and how to design trials with endpoints capable of detecting a real effect against a rapidly progressive disease. Authoritative reviews of γδ immunotherapy converge on the same sober assessment—biological promise tempered by a thin clinical evidence base [102,111,112,113,114]—and glioblastoma, with its formidable defences, will test that promise more severely than most indications.

## 10. Authors’ Perspective and Future Development Strategy

Scope and status of this section: The nine sections above summarise published evidence and are intended to be verifiable against the cited sources. What follows differs in kind: it is the considered opinion of the authors about how the field should proceed, and it is offered as such. Three of us are affiliated with companies active in cell therapy, in the roles set out in the Conflicts of Interest statement; although this review is independent of any commercial programme, describes no proprietary product, received no funding and was not reviewed by any company before submission, readers are entitled to weigh the recommendations below in that light. We have therefore kept the two registers separate: the statements in Section 1, Section 2, Section 3, Section 4, Section 5, Section 6, Section 7, Section 8 and Section 9 rest on citations, whereas the priorities set out here are judgements about which of several defensible paths is likely to be most productive, and reasonable investigators may disagree with them. No recommendation below refers to, or is intended to advance, any product in which the authors have an interest.

Drawing the foregoing evidence together, we offer a considered view of how the field might most productively advance. Our central contention is that the binding constraint on progress is no longer the biological rationale, which is sound and reproducible, but the absence of human data on the parameters that determine whether the rationale translates: how many γδ cells reach the tumour, how long they persist, and under what conditions they remain cytotoxic. Development strategy should be organised around answering those questions rather than around generating further confirmatory cytotoxicity data.

A first priority—in our view directly entailed by the evidentiary gaps documented in Section 5.3, Section 7.2 and Section 7.5 rather than a matter of preference—is to make early-phase trials mechanistically informative. Every study should embed quantitative cell-tracking and serial pharmacodynamic sampling, so that a disappointing clinical result can be attributed to its true cause—failed trafficking, failed persistence or failed function—each of which implies a different remedy [57,58]. Candidate predictive biomarkers, in particular tumour NKG2D-ligand and CD155 expression and the patient’s baseline immune status, should be measured prospectively rather than reconstructed retrospectively [29,53,56]. Without this correlative scaffolding, even a positive trial would leave the field unable to iterate rationally.

A second priority is the deliberate choice of effector subset and engineering level. The Vγ9Vδ2 subset offers ready expandability and phosphoantigen-driven synergy with genotoxic therapy, whereas Vδ1 cells offer tissue residence and a track record as an engineering substrate [15,31,40]; rather than treating these as competitors, we favour parallel evaluation, since the optimal choice may depend on whether the goal is repeated locoregional dosing or a persistent engineered product. On engineering, the drug-resistant-immunotherapy concept is, in our assessment, the most de-risked near-term path because it integrates with the existing standard of care rather than displacing it [59,60,73], but each added modification—chemoresistance, cytokine armouring such as membrane-bound interleukin-15, or a chimeric antigen receptor—trades regulatory and manufacturing simplicity for potency, and that trade should be made explicitly and tested, not assumed [40,68]; the options and their evidential status are set out in Table 6.

A third priority concerns delivery and dosing. We regard allogeneic, off-the-shelf manufacturing combined with locoregional, repeated administration as the most realistic practical model for a recurrent, rapidly progressive disease [15,68], while acknowledging that host rejection of allogeneic cells and uncertain intratumoural migration remain unresolved and may ultimately favour engineered persistence over simple re-dosing. Finally, trial design should be honest about endpoints: given the infiltrative nature of glioblastoma and the confounding of imaging by treatment effect, early studies are better powered on pharmacodynamic and progression-based endpoints with rigorous correlative science than on overall survival, which an underpowered phase 1 cannot credibly address [93,94]. The four registered trials summarised in Table 5 will, over the next several years, begin to supply the human data the field lacks; in our view their correlative outputs will matter as much as their efficacy signals. We advance these positions as a reasoned perspective, not as established fact, and they should be weighed as such.

## 11. Conclusions

γδ T cells bring to glioblastoma a recognition logic that is well matched to the tumour’s principal evasion strategies. They detect malignancy through MHC-independent, antigen-agnostic cues—stress-induced NKG2D and DNAM-1 ligands and phosphoantigen-driven TCR engagement—that are difficult for a heterogeneous, antigen-shedding tumour to escape; they kill stem-like cells implicated in recurrence; they can be expanded from healthy donors for allogeneic, off-the-shelf use with little expectation of graft-versus-host disease; and they cooperate with, rather than oppose, the genotoxic standard of care. These are substantive and reproducible advantages, demonstrated across in vitro systems, animal models and the opening stages of clinical investigation.

Yet the same review that documents these strengths must state plainly that γδ T-cell therapy for glioblastoma remains investigational. The efficacy evidence is overwhelmingly preclinical; the available clinical data establish feasibility and preliminary safety rather than benefit; and serious barriers—the suppressive microenvironment, limited persistence, uncertain trafficking, donor and manufacturing variability, unsettled potency assays and the intrinsic difficulty of glioblastoma trials—stand between a coherent rationale and a proven therapy. The appropriate conclusion is neither dismissal nor advocacy. γδ T cells are a biologically credible and mechanistically distinctive platform whose value in glioblastoma will be settled by further mechanistic study and, decisively, by adequately powered clinical trials with rigorous correlative endpoints. Until those data exist, the strategy should be advanced with disciplined optimism and described as what it is: promising, but unproven.

## Figures and Tables

**Table 1:** Biological characteristics of major human γδ T-cell subsets relevant to glioblastoma immunotherapy. Properties are generalised from the cited literature and simplified for comparison.

Feature	Vγ9Vδ2	Vδ1	Other (e.g., Vδ3)
Main location	Peripheral blood (dominant)	Epithelia, gut, dermis; blood after differentiation	Blood, liver, gut (minor)
Principal activation	Phosphoantigens via BTN3A1/BTN2A1 (inside-out)	Stress self-ligands; CD1/lipid; adaptive-like	Stress ligands; incompletely defined
MHC restriction	None	None	None
Innate receptors	NKG2D, DNAM-1	NKG2D, DNAM-1, NKp30/44 (variable)	NKG2D (variable)
Ex vivo expansion	Robust with zoledronate + IL-2	Feasible; subset-dependent protocols	Limited/specialised
Off-the-shelf suitability	High (allogeneic, low GvHD)	High; favoured for engineering	Under investigation
Relevance to GBM	Most studied; phosphoantigen + NKG2D recognition of glioma	Tissue tropism; CAR-engineering substrate	Hypothesis-generating only

BTN, butyrophilin; GBM, glioblastoma; GvHD, graft-versus-host disease; IL-2, interleukin-2; MHC, major histocompatibility complex; NKG2D, natural killer group 2 member D.

**Table 2:** Principal mechanisms of γδ T-cell anti-glioblastoma activity and their supporting evidence.

Mechanism	Receptor/Effector	Target on Glioma	Nature of Evidence
Stress-ligand recognition	NKG2D	MICA/MICB, ULBPs on tumour and glioma stem-like cells	Glioma lines and primary GSCs; sheddase modulation [44,51,52,53]
Nectin-axis recognition	DNAM-1 (CD226)	CD155, CD112	Mechanistic in γδ; CD155 dependence shown in AML [45,46]
Metabolic sensing	Vγ9Vδ2 TCR via BTN2A1/BTN3A1	Phosphoantigen accumulation	Established in tumours; aminobisphosphonate enhancement [32,33,36,38]
Direct cytotoxicity	Perforin/granzyme; TRAIL	Tumour and cancer stem-like cells	γδ killing of cancer stem cells [47,48]
Immune cross-talk	IFN-γ, TNF; antigen presentation	Myeloid compartment; αβ T cells	Reviewed mechanism; unproven in GBM in vivo [13,14]
Activity vs. stem-like cells	NKG2D + TCR	NKG2D-ligand-bearing GSCs	Ligand expression on GSCs; γδ anti-CSC activity [48,53]

AML, acute myeloid leukaemia; BTN, butyrophilin; CSC, cancer stem cell; GSC, glioma stem-like cell; IFN-γ, interferon-γ; TCR, T-cell receptor; TNF, tumour necrosis factor; TRAIL, TNF-related apoptosis-inducing ligand; ULBP, UL16-binding protein.

**Table 4:** Comparison of cell-based immunotherapy platforms for glioblastoma. Entries generalise platform properties from the cited literature and are necessarily simplified; clinical maturity refers specifically to glioblastoma/high-grade glioma.

Platform	Target Dependence	MHC Restriction	Off-the-Shelf Feasibility	Allogeneic Gvhd Risk	GBM Clinical Maturity
αβ CAR-T	Single defined antigen	MHC-independent	Only if gene-edited	High unless edited	Early-phase; responses without survival benefit [71,72]
TCR-engineered αβ T	Defined peptide–MHC	MHC-restricted	Only if edited	High unless edited	Minimal in CNS [95,96]
TIL	Polyclonal endogenous	MHC-restricted	No (autologous)	n/a	Limited by exhausted TIL pool [97,98]
NK/CAR-NK	Innate ± CAR	MHC-independent	High	Low	Preclinical/early in glioma [100,101]
Vγ9Vδ2 γδ T	Antigen-agnostic + innate	MHC-independent	High	Low/minimal	Preclinical + early-phase [52,53,54,55,57,64,65,73]
Vδ1 γδ T (incl. CAR)	Innate ± CAR	MHC-independent	High	Low/minimal	Engineering-stage [40]

CAR, chimeric antigen receptor; CNS, central nervous system; GBM, glioblastoma; MHC, major histocompatibility complex; NK, natural killer; TCR, T-cell receptor; TIL, tumour-infiltrating lymphocyte.

**Table 5:** Current registered clinical trials of γδ T-cell therapy in glioblastoma/high-grade glioma. Compiled from ClinicalTrials.gov records; status as of early 2026. Inclusion is descriptive and not an endorsement.

Trial/Product	NCT (Phase)	Sponsor (Country)	γδ Product	Population/Combination	Status (Early 2026)
INB-200 (DeltEx DRI)	NCT04165941 (1)	Univ. of Alabama at Birmingham, USA	Autologous, MGMT-modified (drug-resistant); intracranial	Newly diagnosed IDH-wildtype GBM; + maintenance TMZ	Active, not recruiting (fully enrolled)
INB-400 (DeltEx DRI)	NCT05664243 (1b/2)	IN8bio Inc., USA	Allogeneic or autologous, MGMT-modified; intracranial	Newly diagnosed & recurrent GBM; + maintenance TMZ	Active, not recruiting (enrolment paused 2024)
Allogeneic gene-edited γδ	NCT07144735 (early phase 1)	Peking University Third Hospital, China	Allogeneic, gene-edited, off-the-shelf; locoregional	Recurrent/progressive GBM; single-agent	Recruiting
CAR001 (allogeneic CAR-γδ)	NCT06150885 (1/2a)	Ever Supreme Bio Technology, Taiwan	Allogeneic CAR-γδ; intravenous	R/R solid tumours (GBM in expansion cohort); monotherapy	Recruiting

CAR, chimeric antigen receptor; DRI, drug-resistant immunotherapy; GBM, glioblastoma; IDH, isocitrate dehydrogenase; MGMT, O6-methylguanine-DNA methyltransferase; R/R, relapsed/refractory; TMZ, temozolomide. Only the IN8bio programme (INB-200/INB-400) has reported clinical data. The phase 1 INB-200 study, previously available only in abstract form [65,104], has now been reported in full [67]; INB-400 data remain at conference-abstract level [66]. No randomised comparison and no controlled efficacy outcome is available for any γδ product in glioblastoma; the single-arm survival figures reported for INB-200 cannot be read as evidence of benefit. CAR001 is a pan-solid-tumour basket that includes GBM in an expansion cohort rather than a glioblastoma-dedicated study.

**Table 6:** Candidate engineering and pharmacological strategies to sustain γδ T-cell function in glioblastoma, the deficit each addresses and the evidence behind it. Section numbers refer to the discussion of the corresponding deficit in this review. Much of the supporting evidence derives from αβ T-cell engineering or from non-CNS models; entries are candidates for evaluation rather than validated components of a γδ product.

Strategy	Deficit Addressed (Section)	Evidence Base	Principal Cost or Caveat
IL-15-containing expansion (non-genetic)	Low cytotoxic granule content; loss of function under hypoxia (7.2)	Higher perforin, granzyme B, granulysin and T-bet; killing retained under hypoxia [83]	Readily adoptable; effect on in vivo persistence unaddressed
Cell-intrinsic cytokine armouring (e.g., IL-15 secretion)	Short persistence of unmodified products (7.1)	Vδ1 CAR product secreting soluble IL-15 [40]; allogeneic CAR-Vγ9Vδ2 [105]	Genetic modification; toxicity and regulatory burden; untested intracranially
MGMT-mediated alkylator resistance (DRI)	Product exposed to concurrent TMZ (6.3)	Preclinical efficacy [59,60]; clinical feasibility and safety in phase 1 [67]	Most de-risked path; adds gene modification; efficacy unproven
TGF-β pathway disruption (dominant-negative receptor)	Suppressive tumour microenvironment (7.1)	Restores function in antigen-specific αβ T cells [106]	Not demonstrated in γδ cells
Relief of tumour hypoxia	Protein kinase A-driven repression of NKG2D (7.2)	Restored NKG2D and γδ antitumour function in brain tumour models [25]	Clinical means of achieving it in GBM unproven
Adenosine-axis blockade (CD73/A2A)	Metabolic and purinergic suppression (7.2)	CD73 inhibition constrains GBM growth and reshapes microglia in mice [107]	γδ-specific benefit untested
NKG2D-ligand induction (TMZ + PARP inhibitor; ADAM10/17 blockade)	Ligand density and shedding (4.1)	ULBP1 induction with enhanced γδ killing [56]; sheddase inhibition [52]	In vitro and preclinical; combination toxicity undefined
Scheduling with radiotherapy	Trafficking and effector function (7.5)	Synergy of NKG2D-directed T cells with subtherapeutic RT in immunocompetent glioma [108]	Demonstrated for an αβ CAR platform; γδ equivalent untested
STING agonism ± RT	Immune-desert microenvironment; BBB access (7.5)	Survival benefit and transient BBB opening preclinically [109,110]	May recruit IL-17-producing γδ cells and promote radioresistance [84]
Phenotype control and IL-17-axis management	γδ17 polarisation in the irradiated brain (7.3)	Microglia-driven IL-17 polarisation [26]; pro-tumour γδ17 [49,50,84]	Requires IL-17 release testing; no clinical precedent
Editing for allogeneic persistence	Host-versus-graft rejection (7.1)	Strategies developed for allogeneic αβ platforms [68]	Adds substantial modification to an otherwise simple product

BBB, blood–brain barrier; CAR, chimeric antigen receptor; DRI, drug-resistant immunotherapy; GBM, glioblastoma; IL, interleukin; MGMT, O6-methylguanine-DNA methyltransferase; NKG2D, natural killer group 2 member D; PARP, poly(ADP-ribose) polymerase; RT, radiotherapy; STING, stimulator of interferon genes; TGF-β, transforming growth factor-β; TMZ, temozolomide.

## Data Availability

No new data were created or analysed in this study. Data sharing is not applicable to this article.

## Abbreviations

The following abbreviations are used in this manuscript:

ADAM	A disintegrin and metalloproteinase
BBB	Blood–brain barrier
BTN	Butyrophilin
CAR	Chimeric antigen receptor
CCL20	C-C motif chemokine ligand 20
cGAS	Cyclic GMP-AMP synthase
CNS	Central nervous system
CRS	Cytokine release syndrome
DNAM-1	DNAX accessory molecule 1
DRI	Drug-resistant immunotherapy
EGFRvIII	Epidermal growth factor receptor variant III
GBM	Glioblastoma
GSC	Glioma stem-like cell
GvHD	Graft-versus-host disease
HLA	Human leukocyte antigen
ICANS	Immune effector cell-associated neurotoxicity syndrome
IFN-γ	Interferon-γ
IL	Interleukin
MDSC	Myeloid-derived suppressor cell
MGMT	O6-methylguanine-DNA methyltransferase
MHC	Major histocompatibility complex
MICA/B	MHC class I-related chain A/B
NK	Natural killer
NKG2D	Natural killer group 2 member D
OS	Overall survival
PARP	Poly(ADP-ribose) polymerase
PD-1	Programmed cell death protein 1
PFS	Progression-free survival
PKA	Protein kinase A
STING	Stimulator of interferon genes
TCR	T-cell receptor
TGF-β	Transforming growth factor-β
TIL	Tumour-infiltrating lymphocyte
TLR	Toll-like receptor
TMZ	Temozolomide
TNF	Tumour necrosis factor
TOX	Thymocyte selection-associated high mobility group box protein
TRAIL	TNF-related apoptosis-inducing ligand
ULBP	UL16-binding protein
WHO	World Health Organization
